# Model improvement and future projection of permafrost processes in a global land surface model

**DOI:** 10.1186/s40645-020-00380-w

**Published:** 2020-11-13

**Authors:** Tokuta Yokohata, Kazuyuki Saito, Kumiko Takata, Tomoko Nitta, Yusuke Satoh, Tomohiro Hajima, Tetsuo Sueyoshi, Go Iwahana

**Affiliations:** 1 Center for Global Environmental Research, National Institute for Environmental Studies, 16-2 Onogawa, Tsukuba, 305-8506 Japan; 2 Research Institute for Global Change, Japan Agency for Marine-Earth Science and Technology, 3173-25 Showa-machi, Yokohama, 236-0001 Japan; 3 School of Life and Environmental Science, Azabu University, 1-17-71 Fuchinobe, Chuo-ku, Sagamihara-shi, Kanagawa 252-5201 Japan; 4 Institute of Industrial Science, The University of Tokyo, 5-1-5 Kashiwanoha, Kashiwa, Chiba, 277-8574 Japan; 5 International Affairs and Research Development Office, National Institute for Polar Research, 10-3 Midori-cho, Tachikawa, 190-8518 Japan; 6 International Arctic Research Center, The University of Alaska Fairbanks, 2160 Koyukuk Dr, Fairbanks, AK 99775-7340 USA

**Keywords:** Permafrost degradation, Global climate model, Climate change

## Abstract

To date, the treatment of permafrost in global climate models has been simplified due to the prevailing uncertainties in the processes involving frozen ground. In this study, we improved the modeling of permafrost processes in a state-of-the-art climate model by taking into account some of the relevant physical properties of soil such as changes in the thermophysical properties due to soil freezing. As a result, the improved version of the global land surface model was able to reproduce a more realistic permafrost distribution at the southern limit of the permafrost area by increasing the freezing of soil moisture in winter. The improved modeling of permafrost processes also had a significant effect on future projections. Using the conventional formulation, the predicted cumulative reduction of the permafrost area by year 2100 was approximately 60% (40–80% range of uncertainty from a multi-model ensemble) in the RCP8.5 scenario, while with the improved formulation, the reduction was approximately 35% (20–50%). Our results indicate that the improved treatment of permafrost processes in global climate models is important to ensuring more reliable future projections.

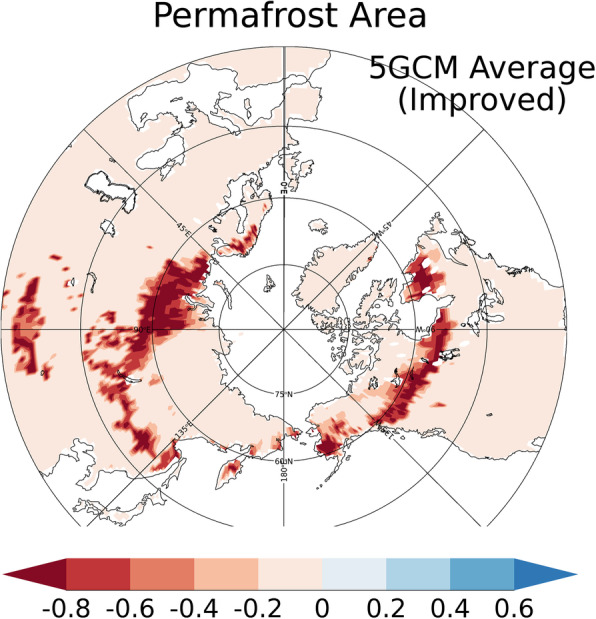

## Introduction

Global warming is expected to produce a thawing of the permafrost (Koven et al. 2013; Slater and Lawrence 2013; Vaks et al. 2013; Koven et al. 2015; McGuire et al. 2018). As a result, large amounts of organic matter confined in the permafrost will decompose, releasing greenhouse gases (GHGs) such as carbon dioxide and methane (Zimov et al. 2006; Schuur et al. 2008; Brown 2013; Schaefer et al. 2014; MacDougall et al. 2015; Schneider von Deimling et al. 2015; MacDougall and Knutti 2016; Steffen et al. 2018; Gasser et al. 2018; McGuire et al. 2018; Kawamiya et al. 2020; Saito et al. 2020; Yokohata et al. 2020). Unfortunately, the permafrost processes represented in current global climate models (GCMs) tend to be rather simplistic because of the significant uncertainties regarding the dynamics of the permafrost due mainly to a lack of observational knowledge (Alexeev et al. 2007; Nicolsky et al. 2007; Lawrence et al. 2008; Rinke et al. 2008; Koven et al. 2009; Gouttevin et al. 2012). Projections of permafrost thawing vary substantially in distribution and magnitude depending on the climate model used (Koven et al. 2013; Slater and Lawrence 2013). This wide variation in projections can be explained by the fact that the rate of degradation of the permafrost is closely related to a variety of factors in the climate system, including changes in the surface air temperature, precipitation, and evaporation in the high latitudes, all of which involve a great deal of uncertainty.

In this study, we improved the modeling of permafrost processes in the global land surface model MATSIRO (Minimal Advanced Treatments of Surface Interaction and Runoff, Nitta et al. 2014), which is used in the global climate model MIROC (Model for Interdisciplinary Research on Climate, Watanabe et al. 2010). We conducted historical simulations by using both conventional schemes and our proposed scheme and compared simulation results to observations. We also conducted future simulations using the two schemes and assessed the impact of our model improvement, as well as the sources of uncertainty in future simulations.

Previous research (Lawrence et al. 2008; Saito 2008a; Saito 2008b; Saito 2011; Chadburn et al. 2015; Melton et al. 2019) has recognized the importance of taking into account changes in thermodynamic quantities due to the freezing of soil moisture and the physical characteristics of the organic layer near the ground surface, and presence of unfrozen water in order to obtain more realistic permafrost distributions in simulations using global climate models. However, this previous research with MATSIRO (Saito 2008a, 2008b, 2011) involved only historical or ideal future simulations at particular points where observational data were available (e.g., Utqiagvik, formerly known as Barrow) or conducted only historical global simulations. Consequently, neither the impact of the proposed improvements in the modeling of the physical properties of permafrost processes nor the uncertainties in the projection of future atmospheric forcing were evaluated.

This study directly addresses both issues. We first evaluate the effects of the improvements in the modeling of the physical processes related to permafrost on the reproducibility of the observed permafrost distribution and analyze the effect of these improvements on the future projection of the permafrost distribution. We also assess the effect of the uncertainties associate with the atmospheric forcings on the simulations of the permafrost distributions. For this purpose, we apply atmospheric forcings generated by bias-corrected global climate models to the global land surface model and then perform multiple future simulations.

## Methods

### Improvement of permafrost processes in a global climate model

In this study, the proposed improvements in the modeling of the permafrost processes include the following considerations:

(1) Consideration of the heat capacity and thermal conductivity of frozen soil.

(2) Consideration of the organic layer near the surface in the high-latitude Taiga and Tundra regions.

(3) Consideration of unfrozen water in regions where the ground temperature is below 0 °C

The details of consideration (1) and (3) are provided in Saito (2008a). In brief, in the conventional scheme, all soil moisture is treated as liquid water when calculating soil heat capacity *C* and thermal conductivity *K* (Takata et al. 2003). However, frozen water has a smaller heat capacity and greater thermal conductivity than liquid water. To reflect this, in the improved scheme that we propose, when water is frozen the heat capacity is smaller and the thermal conductivity is greater than is the case in the conventional scheme (Saito 2008a, 2008b). Similarly, unfrozen water content is present but decreases exponentially with the subfreezing temperature where the exponent coefficient is predetermined by the soil types (e.g., sand, silt, and clay).

Saito (2011) gives details of the improved treatment of the organic layer as described in consideration (2). In the current study, the soil parameter values are further modified to increase model performance. The land surface model MATSIRO determines the vegetation and soil type for each grid. The six physical properties of soil (volumetric specific heat, thermal conductivity, porosity, saturated matrix potential, saturated hydraulic conductivity, void size distribution index) are then set according to the soil type (Takata et al. 2003; Saito 2011). In this study, we first determined the uncertainty ranges for these soil parameters based on expert judgement, and then perturbed these parameters one by one within the uncertainty ranges. Then, we found a parameter set for which the permafrost area obtained by the land surface model (as illustrated in Fig. 2) is close to the permafrost region estimated from observations by the International Permafrost Association (IPA; Brown et al. 1998).

For both the conventional and improved schemes described here, six vertical layers and a bottom depth of 14 m were used (Nitta et al. 2014). In the conventional scheme, constant physical properties are given according to soil type regardless of the vertical depth (Takata et al. 2003). The definition of soil types and the values for the six physical properties are the same as those in Nitta et al. (2014). In the improved scheme, in high-latitude regions with Tundra vegetation types, the top three layers (0–1 m) are organic layers, the middle two layers (1 m–4 m) are mineral soil layers, and the bottom layer (4–14 m) is the bedrock layer, mainly consisting of rocks as shown in Table 1. In the regions with taiga vegetation type, the top four layers (0–2 m) are organic, the fifth layer (2–4 m) is mineral soil, and the bottom layer (4–14 m) is the bedrock layer (Table 1). The values for the physical parameters are different depending on the type of soil layer. In the grids for regions other than the Taiga or Tundra regions, the top five layers are mineral soil layers, and the bottom layer is the bedrock layer. The soil type in the improved scheme is given in the same way as in Saito (2011). However, the values for the six physical properties have been improved. Table 1 summarizes these values for the organic, mineral, and base layers used in the improved and conventional scheme.

**Table 1:** Parameter values for the organic, mineral soil, and base layers used in the improved scheme. The values for the mineral layers are dependent on the soil type. Here, the values of the mineral layers for the “sand” soil type are shown. The layers for the organic (moss and soil), mineral soil, and bed lock layer for the improved and conventional schemes are also shown

	Organic (moss)	Organic (soil)	Mineral	Bedrock
Layers in the improved scheme (Tundra)	Top layer (0–5 cm)	2nd–3rd layers (5–1 m)	4th–5th layers (1–4 m)	Bottom layer (4–14 m)
Layers in the improved scheme (Taiga)	Top two layers (0–25 cm)	3rd–4th layers (25 –2m)	5^th^ layer (2–4 m)	Bottom layer (4–14 m)
Layers in the original scheme	–	–	All layers (0–14 m)	–
Volumetric specific heat [MJ/m^3^ K]	2.5	2.5	2.1	2.3
Thermal conductivity [W/m K]	0.12	0.25	2.0	3.0
Saturated soil moisture (porosity) [−]	0.8	0.7	0.48	0.3
Saturated hydraulic conductivity 10^−6^ [m/s]	15	2.0	1.71	5.0
Saturated hydraulic potential [m]	− 0.085	− 0.12	− 0.0563	− 0.300
Void size distribution index [−]	2.1	4.0	3.6	3.3

### Experimental settings

To investigate the effect of our improvements in the modeling of the permafrost processes, we performed offline simulations using a global land surface model MATSIRO (Nitta et al. 2014). In these simulations, atmosphere-ocean processes in the global climate model MIROC (Watanabe et al. 2010) were not calculated but were given as an atmospheric forcing to drive the land surface model. For the atmospheric forcing, we used the data provided by the Inter-Sectoral Impact Model Inter-comparison Project, Phase 1 (ISIMIP1, Hempel et al. 2013). In the ISIMIP1 forcing, atmospheric variables were calculated by five GCMs (GFDL-ES2M, Dunne et al. 2012; HadGEM2-ES, Jones et al. 2011; IPSL-CM5A-LR, Dufresne et al. 2013; Nor-ESM, Bentsen et al. 2013; MIROC-ESM-CHEM, Watanabe et al. 2011), and model bias was corrected in the historical and future simulations.

**Fig. 1:**
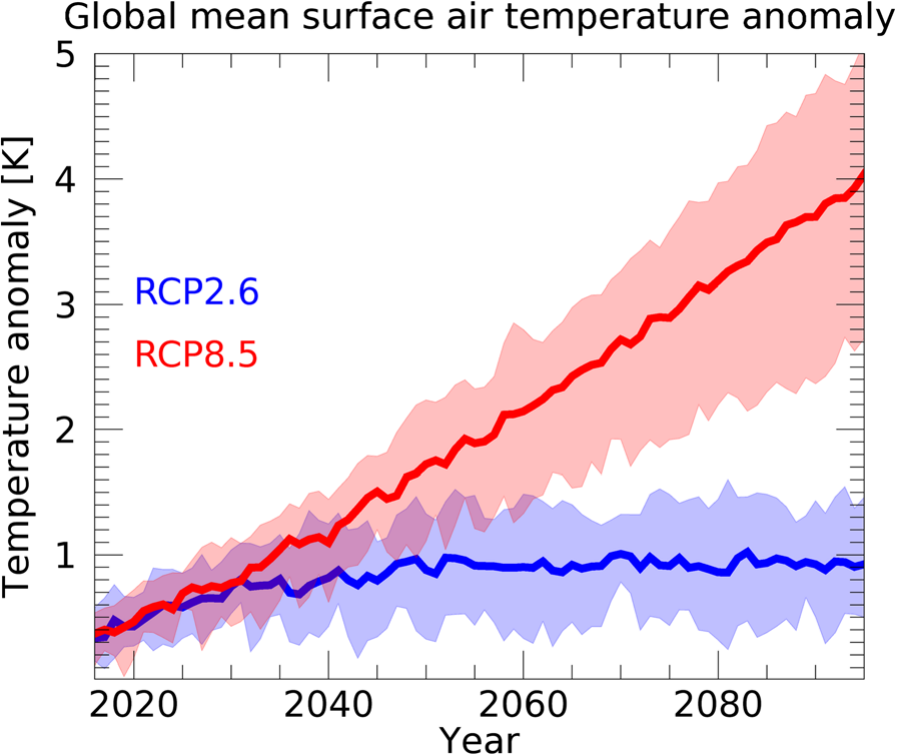
Time sequence of the global mean surface air temperature anomaly given as atmospheric forcing to the land surface model. The width of the lines represents the maximum and minimum values of the simulated results forced by the five GCMs; the average value is represented by the bold lines. The global mean surface air temperature under RCP2.6 (blue) and RCP8.5 (red) scenarios is shown. Anomaly of surface air temperature is calculated with respect to the 1995–2014 average

In the present study, we performed our historical and future simulations by giving the atmospheric forcings of the five GCMs to MATSIRO. The climate scenarios in the future simulations were based on Representative Concentration Pathways (RCP, van Vuuren et al. 2011); specifically, we used the low-carbon scenario (RCP2.6) and the high-warming business-as-usual scenario (RCP8.5). The global average of the surface air temperature anomaly given as the atmospheric forcing for RCP2.6 and RCP8.5 is shown in Fig. 1.

## Results and discussion

### Comparison of model simulations with observations

**Fig. 2:**
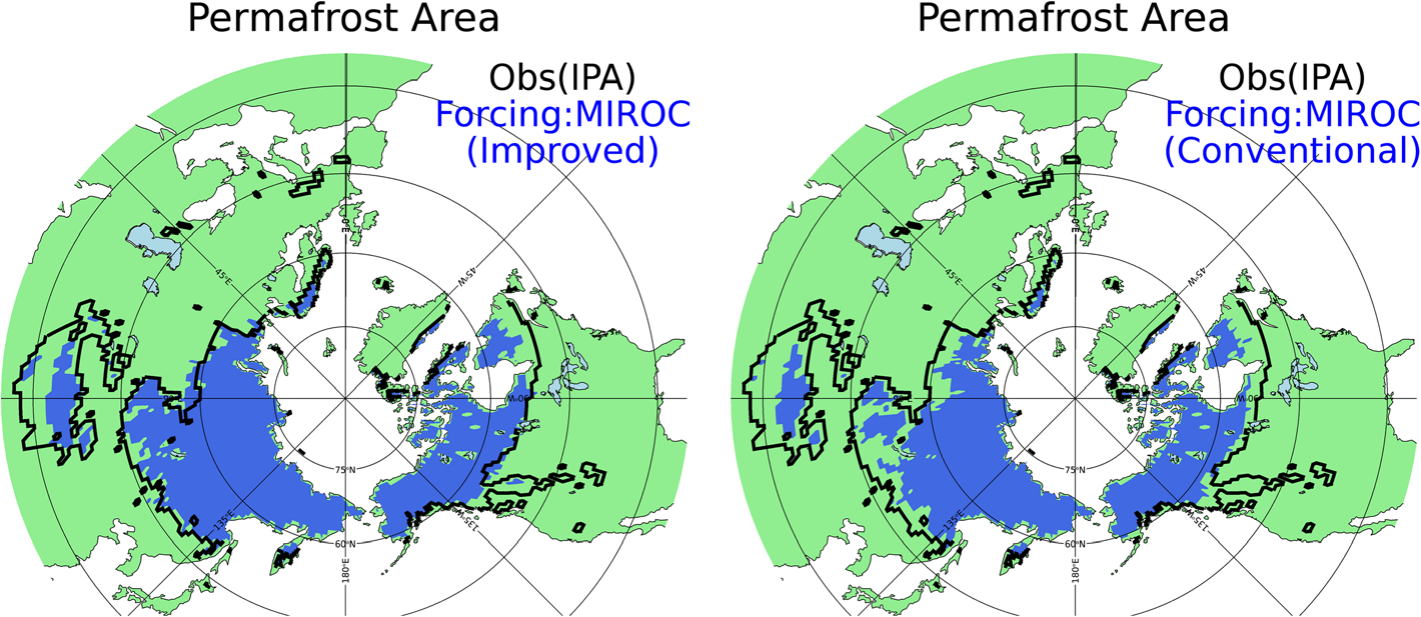
Permafrost area of the historical model simulation (blue) and observation (outlined in black). The historical simulation was performed with the MIROC atmospheric forcing. The permafrost area is determined using the soil temperature for the year 2000. The observed permafrost area is from Brown et al. (1998)

Figure 2 shows the permafrost area results obtained by our MATSIRO offline simulation. The permafrost area is defined as the regions where the soil temperature is kept below 0 ^o^C in any of the soil layers throughout the year. In Fig. 2, the permafrost region estimated from observations by the IPA (Brown et al. 1998) is outlined in black and compared to the areas estimated in the model simulations. In the MATSIRO offline simulation, the results of the GCM simulations were used as the atmospheric forcing (Section 2.2). As shown in Fig. 2, the permafrost area estimated with the improved scheme is larger than that in the conventional scheme, especially in southern Siberia and southern Canada. Importantly, the estimated area under the improved scheme shows better agreement with the observation-based estimate.

Consideration of three additional factors accounts for the superior performance of the improved version. These include (a) the heat insulation effect of the organic layer, (b) the increase in heat conduction in winter due to the presence of ice, and (c) the decrease in ground temperature in winter due to the presence of unfrozen water. In summer, factor (a) suppresses the soil temperature rise in the improved version compared to the conventional version. On the other hand, in winter, (b) and (c) promote a decrease in soil temperature.

**Fig. 3:**
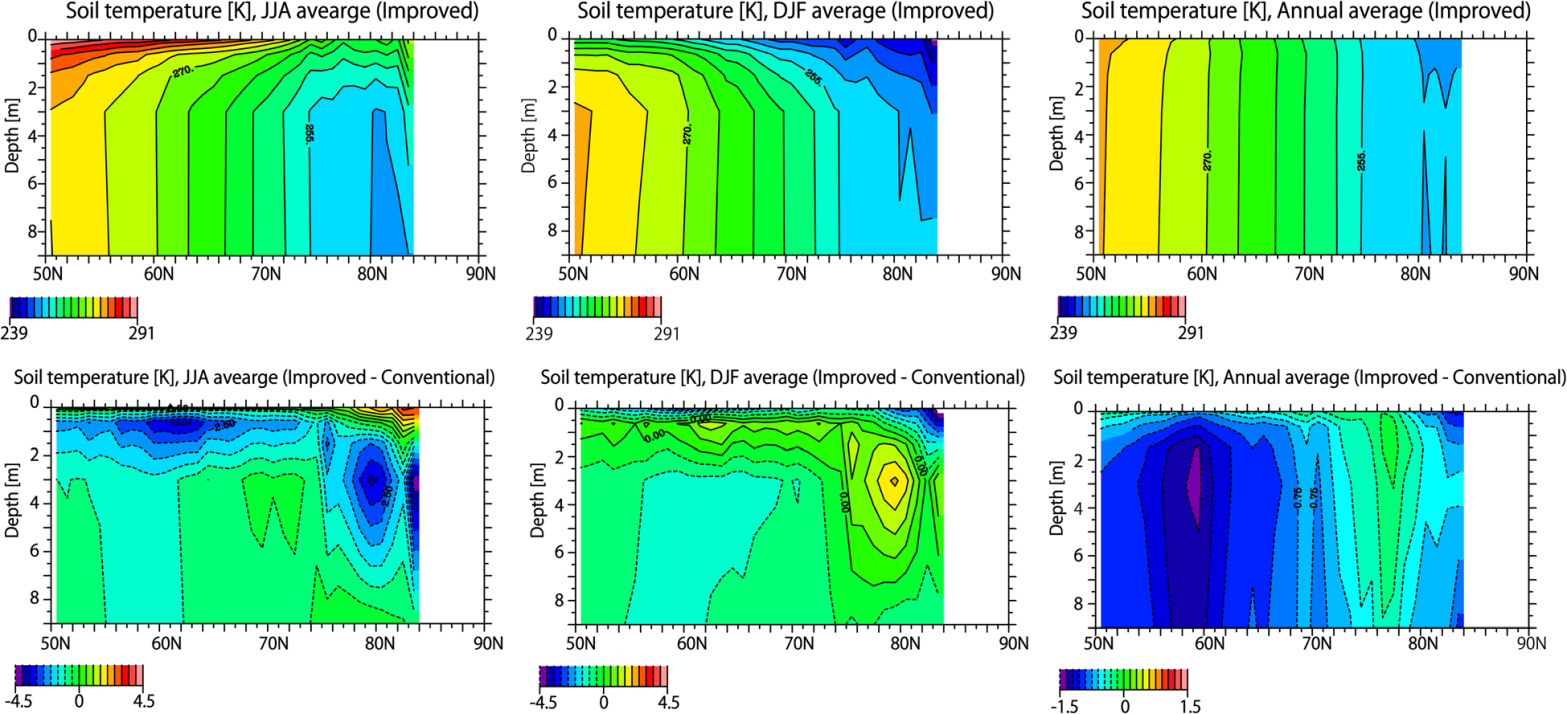
Latitudinal distributions of soil temperature in the historical simulations. Upper panels: the JJA, DJF, and annual average of soil temperature for the year 2000 in the improved version. Lower panels: the same as the upper panels but showing the differences between the improved and conventional versions. Vertical axis represents the soil depth [m], and the longitudinal average over land is shown. The average simulations with the atmospheric forcings of all five GCMs is shown

Figure 3 shows the latitude-depth soil temperature distribution in the present climate. The upper panel of Fig. 3 shows the JJA, DJF, and annual average soil temperature distribution in the improved version. The soil temperature near the surface is higher than in the lower layer in the summer, but lower in winter. The vertical gradient of the soil temperature near the surface (to a depth of approximately 3 m) is large, but becomes smaller with increasing depth. The annual average soil temperature is essentially determined according to latitude. The lower panel of Fig. 3 shows the difference in JJA, DJF, and annual average soil temperature between the improved and conventional versions. In summer, the soil temperature is lower in the improved version due to factor (a), the heat insulation effect of the organic layer. In winter, the soil temperature is higher in places due to the effect of (a), but is lower in many regions due to the effects of (b) and (c). For these reasons, in terms of the annual average value, the soil temperature is lower in the improved version over the entire area. As a consequence, the improved version has a larger distribution of permafrost, as shown in Fig. 2.

**Fig. 4:**
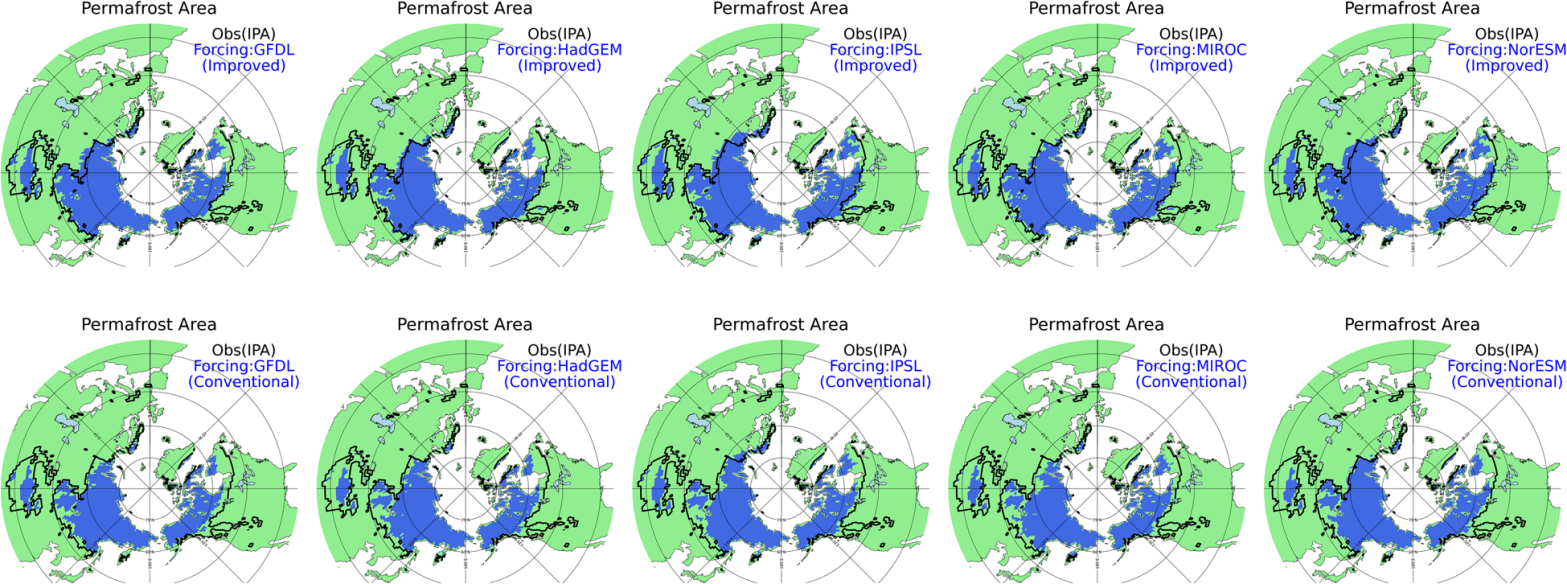
Permafrost area of the historical model simulation (blue) and observation (outlined in black) for the simulations with the atmospheric forcings of all five GCMs (GFDL-ES2M, Dunne et al. 2012; HadGEM2-ES, Jones et al. 2011; IPSL-CM5A-LR, Dufresne et al. 2013; Nor-ESM, Bentsen et al. 2013; MIROC-ESM-CHEM, Watanabe et al. 2011). The upper panel shows the results with the improved scheme; the lower panel shows the results with the conventional scheme

Figure 2 shows the simulation results when the atmospheric forcing of MIROC is given. The permafrost areas forced by all the various GCMs are shown in Fig. 4. The tendency of the permafrost area in the improved scheme to be larger and closer to the observed area than is the case for the conventional scheme is evident in all the simulation results forced by the five GCMs. More specifically, from the eastern part to the western part of Siberia, the improved version reproduces the southern limit of permafrost very well (reproducibility is especially high in GFDL and MIROC). On the other hand, from the eastern part to the western part of Siberia, the conventional version underestimates the permafrost distribution in all the simulations with different atmospheric forcings. Similarly, the improved version reproduces the southern limit of permafrost very well in central North America in all the simulations, while the permafrost area is underestimated in eastern and western Canada and the Himalayan region. This may be related to the fact that the global land surface model has a resolution of 1° and that topographical details cannot be expressed, and thus permafrost is not reproduced adequately in the simulations.

**Fig. 5:**
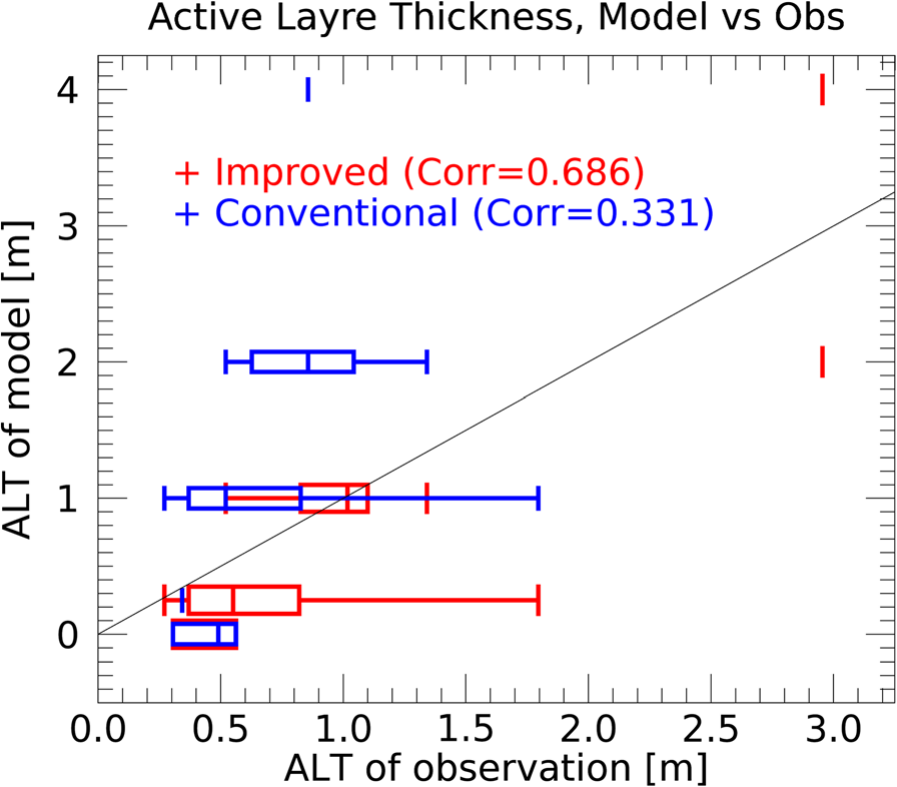
Active layer thickness for model results (vertical axis) versus observed ALT values (horizontal axis). The results of the improved (red) and conventional (blue) schemes are shown. The box and whisker plot indicates the minimum, lower quartile, median, upper quartile, and maximum values of observation. The correlation coefficients for the relationship between the model results and the observed data are also shown

Figure 5 compares the active layer thickness (ALT) in the model simulations with the observed values. We used the observation data obtained by Circumpolar Active Layer Monitoring (CALM) at the same forty sites that were used in Saito (2011). Here, a simulated ALT result in the grid cell closest to a CALM site was chosen. For the calculation of the ALT value from the model simulations, we chose the maximum depth of the model layers each year at which the soil temperature (monthly average) is larger than 0 ^o^C, following the procedure of Koven et al. (2015). The ALT value for the improved scheme is smaller than that for the conventional scheme, which is consistent with the results of the permafrost area comparison shown in Fig. 2. The correlation between the ALT values in the model simulations and the observed values in the improved and conventional schemes is 0.688 and 0.331, respectively. Thus, the simulated ALT values for the improved scheme appear to be in better agreement with the observations than those in the conventional scheme. Given these results, we concluded that a more realistic treatment of the soil freezing processes can improve the reproducibility of the permafrost area in the model simulations.

## Future simulations

**Fig. 6:**
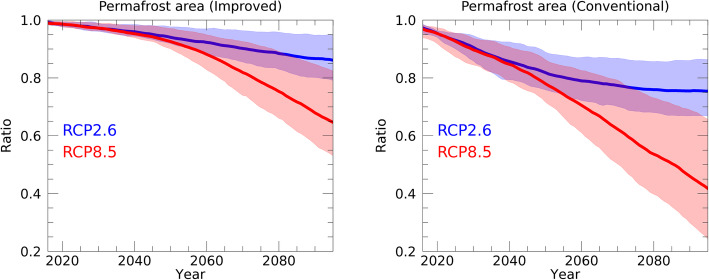
Changes in the total permafrost area across the world as projected by the study’s future simulations. The width of the line represents the maximum and minimum values of the simulated results forced by the five GCMs; the average value is represented by the bold lines. The simulations with the improved (left) and conventional (right) schemes under RCP2.6 (blue) and RCP8.5 (red) scenarios are shown. Values on the vertical axis were determined by setting the permafrost area at the initial year (2005) of the future simulations to 1.0

Figure 6 shows future projections of the permafrost area using the improved and conventional schemes. Here, the permafrost area is diagnosed in the same way as in Fig. 2. Overall, the simulated response of the permafrost area to global warming is consistent with the features described above (Figs. 2, 3, 4, and 5). As shown in Fig. 6, the permafrost area decreases more rapidly under the conventional scheme than under the improved scheme. Figure 6 indicates that changes in the way in which the freezing processes are represented in the model greatly influence the model’s projections of the future, as well as its description of the present.

A detailed comparison of the simulated changes in the permafrost area provides additional useful information. In both the improved and conventional schemes, the permafrost area decreases monotonically until the year 2040 (approximately) for both the RCP2.6 and RCP8.5 scenarios. After 2040 (approximately), the reduction rate of the permafrost area slows in the RCP2.6 scenario, while it accelerates in RCP8.5. More specifically, with the improved scheme, in the RCP8.5 scenario, the permafrost area decreases by an average of roughly 35% (average of the ensemble members) by 2100, with a high estimate of 50% (maximum of the ensemble members), while with the conventional scheme, the average decrease is roughly 60%, with a high estimate of 80%.

**Fig. 7:**
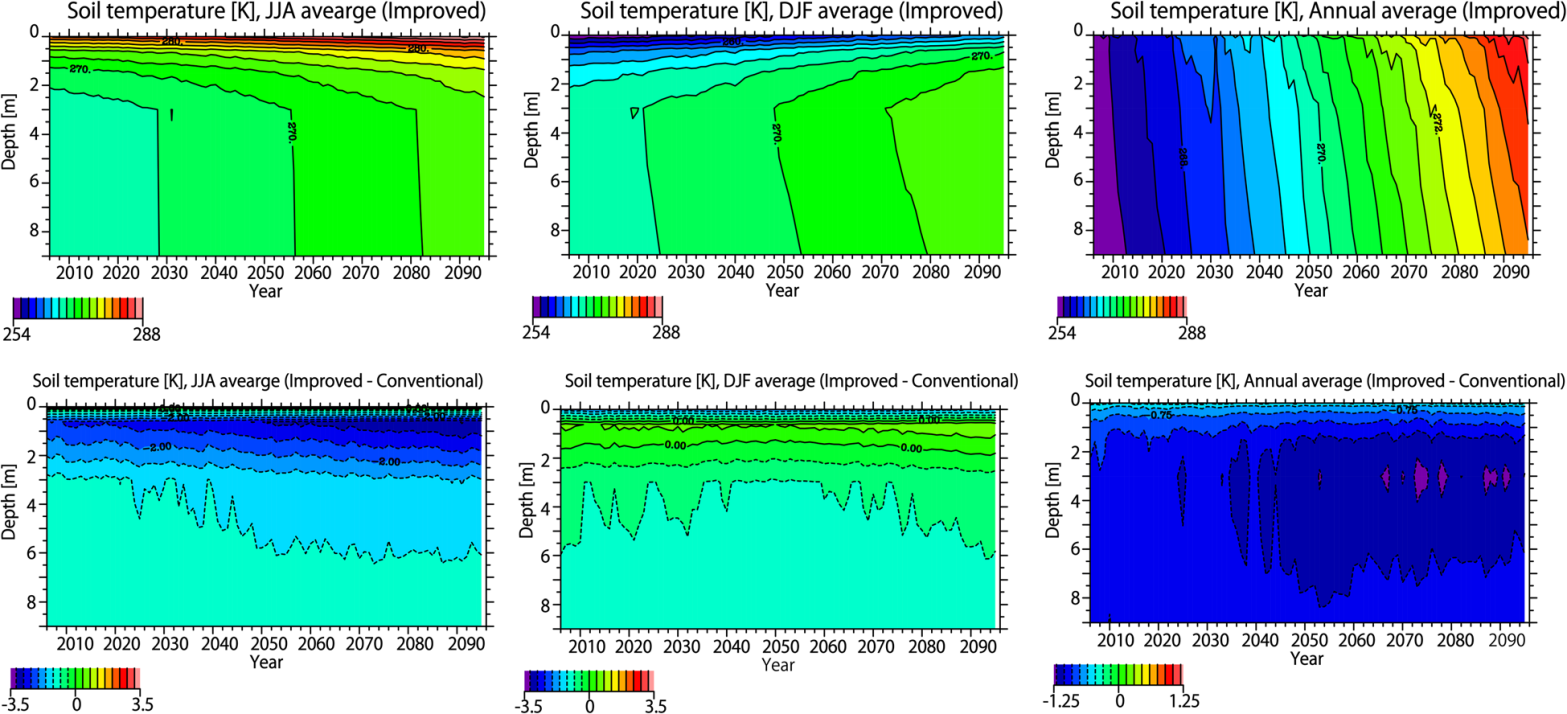
Time sequence of soil temperature. Upper panels: soil temperature for JJA, DJF, and annual average in the improved scheme. Lower panels: The same as upper panels, but showing the differences between improved and conventional scheme. Vertical axis represents the soil depth [m], and the longitudinal average of the soil temperature north of 50° is shown.

In order to investigate the differences in the permafrost distribution between the improved and conventional schemes, the time sequence of vertical distribution of soil temperature is shown in Fig. 7. As described above, the soil temperature near the surface is high in summer and low in winter, and the vertical gradient of soil temperature is small below 3 m. As the air temperature rises through the twenty-first century, the soil temperature increases at all depths. As discussed above, the improved scheme considers (a) the organic layer near the surface, (b) the thermal conductivity of ice, and (c) unfrozen water. The effect of (a) suppresses the increase in soil temperature especially in summer and the effect of (a) is canceled by that of (b) and (c) in winter; thus, the annual average soil temperature in the improved scheme is lower than that in the conventional scheme over a wider area.

As shown in Fig. 7, the difference in summer soil temperature determines the difference in annual mean soil temperature between the improved and conventional schemes. Due to the effect of (a) in summer, the total heat flux reaching deep in the ground is larger in the conventional scheme than in the improved scheme. Since the difference in soil temperature is determined by the difference in the integrated amount of the heat flux reaching to the ground layers, the difference in soil temperature between the improved and conventional scheme increases with time. For this reason, the amount of permafrost reduction is larger in the conventional scheme than in the improved scheme.

**Fig. 8:**
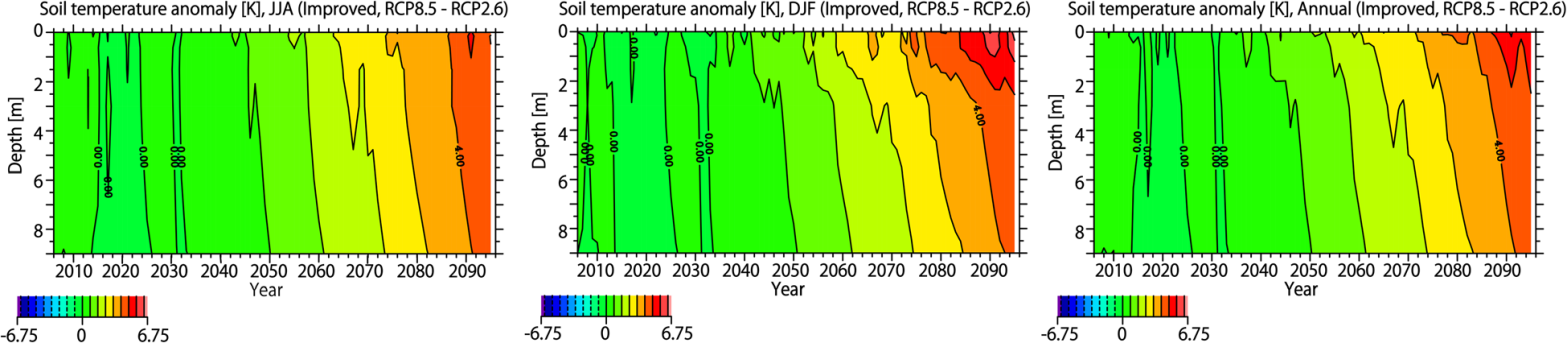
Difference in soil temperature between RCP2.6 and RCP8.5. The time sequences of the vertical distribution of soil temperature for JJA, DJF, and annual average are shown. Vertical axis represents the soil depth [m], and the longitudinal average of the soil temperature north of 50° is shown

Next, the difference in the permafrost change between the RCP2.6 and RCP8.5 scenarios in Fig. 4 is investigated. In this study, the future projection of surface air temperature by five GCMs is applied to the land surface model as the atmospheric forcing (Fig. 1). In the future projection of surface air temperature, the average values of the five GCMs under RCP2.6 and RCP8.5 are nearly the same until around 2030. Changes in the permafrost area (Fig. 4) are a delayed response to changes in surface air temperature, and the changes in permafrost areas in the two scenarios are nearly the same until around 2050 in the improved scheme. Figure 8 shows the difference in the vertical distributions of soil temperature between the RCP2.6 and RCP8.5 scenarios in the improved scheme. As indicated in Fig. 8, a difference in soil temperature between RCP2.6 and RCP8.5 begins to occur near the surface at around 2040, and it takes approximately 10 years to reach the bottom layer. As a result, differences in the permafrost area between RCP2.6 and RCP8.5 will appear roughly 20 to 30 years after the difference in surface air temperature between these scenarios occurs.

**Fig. 9:**
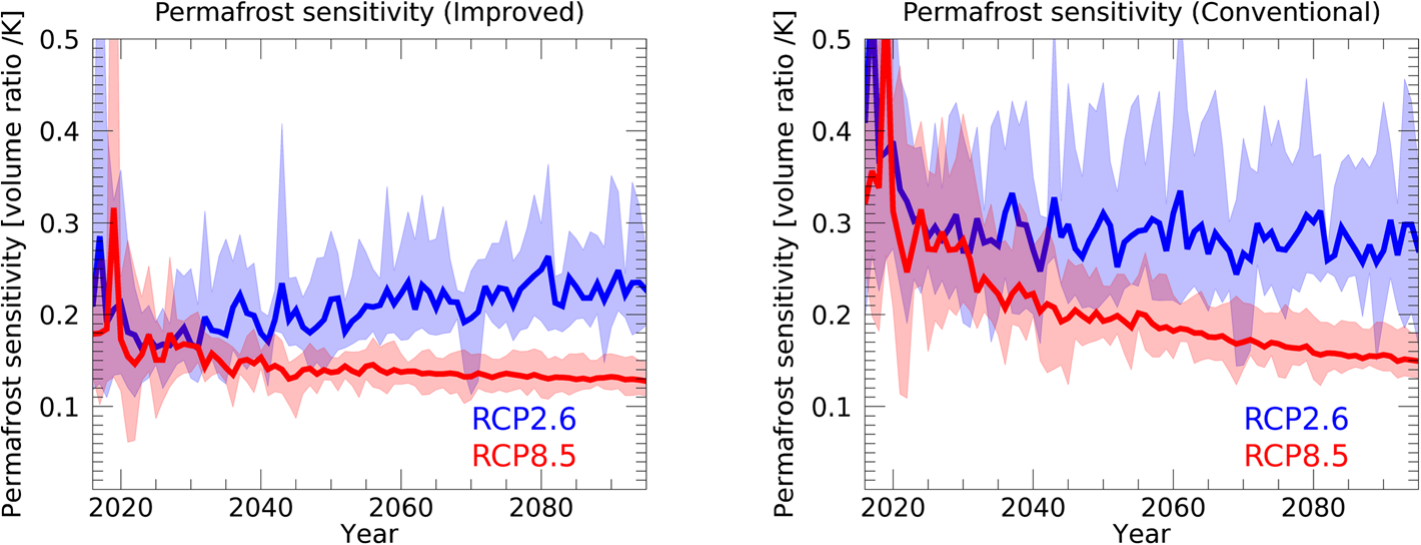
Time sequence of permafrost sensitivity. The change in permafrost volume divided by the change in global mean surface air temperature [ratio/degree] is shown. The width of the colored bands represents the maximum and minimum values of the simulated results forced by the five GCMs; the average value is represented by the bold lines. The simulations with the improved (left) and conventional (right) schemes under RCP2.6 (blue) and RCP8.5 (red) scenarios are shown. Anomaly of permafrost volume and surface air temperature is calculated with respect to the 1995–2014 average.

We also investigated the relationship between permafrost changes and global mean surface air temperature (Fig. 9). Burke et al. (2020) reported that permafrost sensitivity—the change in permafrost volume per degree of global mean surface air temperature change—is 20–30% using CMIP5/CMIP6 data. Although these authors analyzed the uncertainty ranges in multi-model ensembles of CMIPs, the present study could examine the response of a global land surface model by considering the uncertainty in atmospheric model projection. As shown in Fig. 9, permafrost sensitivity is similar for RCP2.6 and RCP8.5 until around 2020. After 2040, a difference emerges, with RCP2.6 showing higher permafrost sensitivity. The reason for this higher sensitivity in RCP2.6 is that, in this scenario, the global mean surface air temperature stabilizes in the latter half of the twenty-first century, which means changes in permafrost volume also stabilize during this time. In contrast, in RCP8.5, the global average temperature continues to rise throughout the century, and thus the response of soil temperature and permafrost volume is not stabilized. With the five atmospheric forcings in the improved scheme, the permafrost sensitivity in RCP2.6 is 0.2–0.3/°C at the end of the current century. On the other hand, the permafrost sensitivity for the RCP8.5 scenario in the improved scheme is 0.11–0.14/°C, which is approximately half that for the RCP2.6 scenario.

**Fig. 10:**
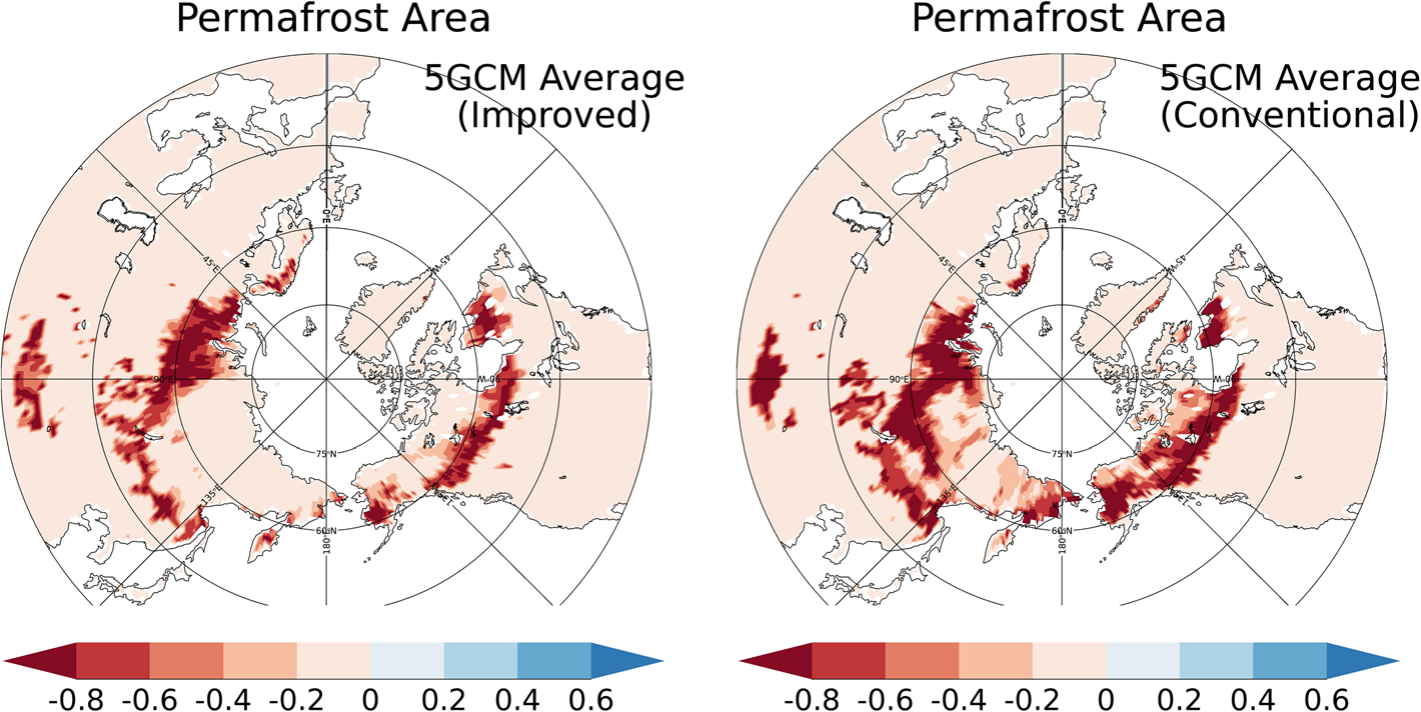
Changes in the permafrost distribution projected by the study’s future projection simulations. The current permafrost area is set at 1.0. Average values for the simulations with atmospheric forcings by the five GCMs are plotted. The difference between the average for 2081–2090 and 2006–2015 under RCP8.5 was calculated

Figure 10 shows the changes in the distribution of the permafrost regions with the improved and conventional schemes. As can be seen in the figure, the permafrost regions estimated with the conventional scheme retreats to higher latitudes than is the case for the improved scheme. On the North American continent, the permafrost regions with the conventional scheme decreases at the higher latitudes over most of the longitudinal band. In Siberia, the permafrost area is reduced around a latitude of 60 °N within longitudes 90–135 °E with the conventional scheme, but not so with the improved scheme (Fig. 10). On the other hand, the permafrost area does not decrease very much within longitudes of 90–135 °E.

**Fig. 11:**
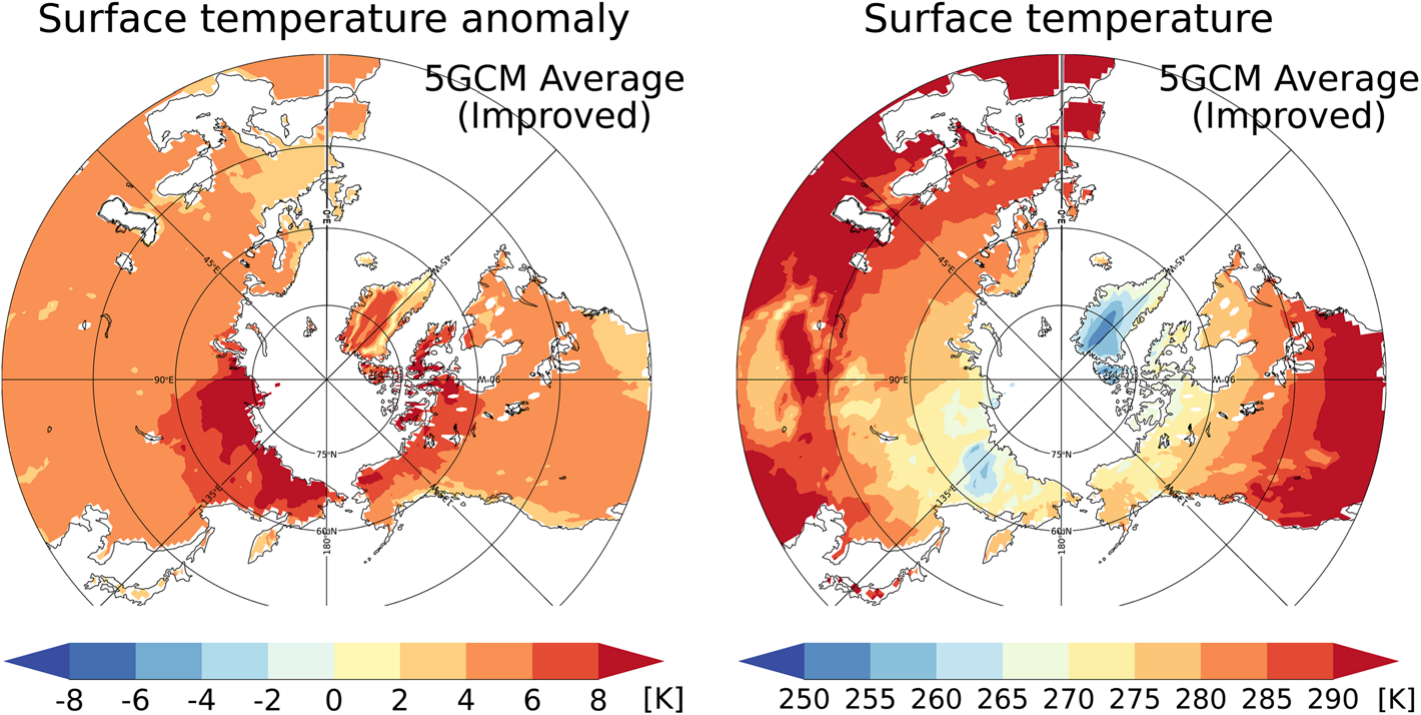
Future projections of soil temperature changes. **a** Changes in the soil temperature (top 5 cm) between 2081–2090 and 2006–2015 [K]; **b** Soil temperature (top 5 cm) averaged over 2081–2090. The average values for the simulations with atmospheric forcings by the five GCMs are plotted

Interestingly, reductions in the permafrost area differ depending on longitude, as shown in Fig. 10. The features of permafrost thawing must be related to the different types of permafrost under the current climate, which varies greatly among different regions due to geological, geographical, and historical reasons. As indicated in Fig. 2, in the eastern part of the Eurasian continent (around longitudes 90–135 °E), permafrost exists in the lower latitudes to roughly 45 °N (owing to the Tibetan Plateau). On the other hand, there is no permafrost area in the western part of the continent (around 0–90 ^o^E) below latitude 60 ^o^N (approximately) because of the temperate marine climate due to the Gulf Stream. These temperate climate conditions make the western part of the Eurasian continent (around 0–90 °E) more prone to permafrost thawing (Fig. 10). Conversely, the permafrost regions in the eastern part of the Eurasian continent (or east Siberia, roughly 90–135 ^o^E) are less likely to be reduced because of the low surface air temperature, even at relatively low latitudes due to elevation. Figure 11 shows the future projections of the near-surface soil temperature (top 5 cm) in the RCP8.5 scenario simulated with the improved scheme. Although the changes in soil temperature are large in the eastern part of the Eurasian continent (90–135 °E, Fig. 11a), the soil temperature remains below 0 °C, even at the end of the twenty-first century (Fig. 11b). In all, these findings confirm the proposition that future changes in the permafrost area are closely related to the features of current climate conditions and permafrost characteristics.

## Conclusions

By refining the process associated with the freezing and thawing of soil moisture—which has been simplified in previous global climate models—the reproducibility of the model used here improved noticeably (Figs. 2 and 4). In general, in order to ensure more reliable future projections, it is critical that a predictive model have the ability to accurately reproduce reality. The analysis in this study showed that the reproducibility of the featured model was enhanced by improving the sophistication of the basic freeze and thaw processes included in the model, particularly soil heat capacity and thermal conductivity. The findings reported here have the potential to greatly improve future projections of the permafrost area, which is an important physical quantity in climate change research.

According to a study evaluating the future projection results produced by the GCMs of the 5th Coupled Model Inter-comparison Project (CMIP5, Koven et al. 2015), the rate of decrease in the permafrost area for the RCP8.5 scenario was 30 to 100%, with a high degree of uncertainty attached to the projections. Koven et al. (2015) used the results of 18 GCMs to quantify the uncertainties. However, the structure of the models (i.e., model parameterization, resolution) is quite different among the various CMIP5 GCMs (i.e., Knutti et al. 2013; Yokohata et al. 2013), making it difficult to precisely identify the causes of the uncertainties in the model projections. On the other hand, the analysis in this study revealed that the thermophysical properties (heat conduction and heat capacity) of the soil greatly affect the reproducibility of the current permafrost distribution (Figs. 2 and 4). Based on our analysis, it seems clear that a realistic treatment of such physical properties (e.g., thermophysical variables) is important to producing more realistic future projections.

**Fig. 12:**
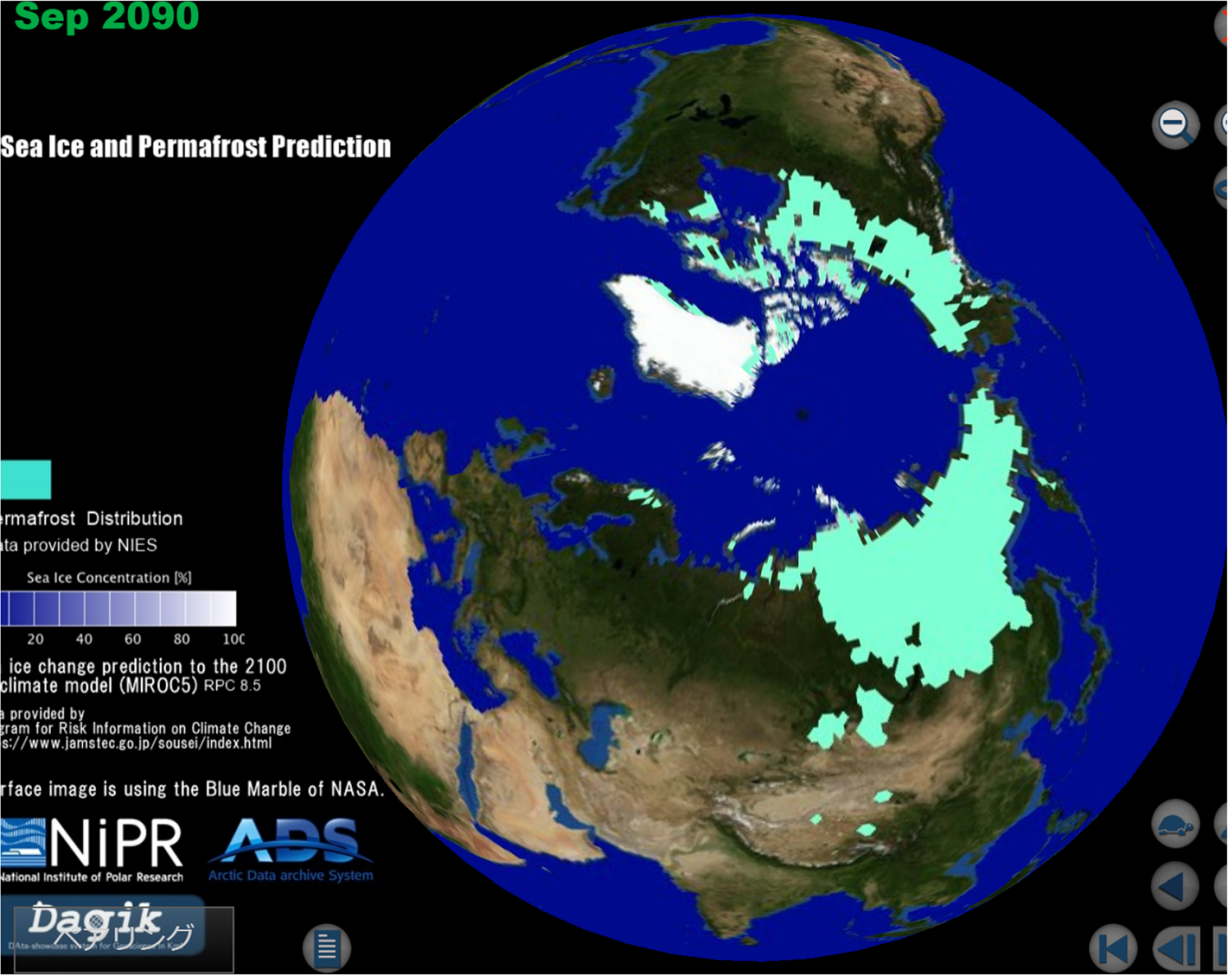
Snapshot of an animation of future projections of the permafrost area based on data from simulations using the improved scheme with the MIROC atmospheric forcing under the RCP8.5 scenario. The animation was generated based on a tool developed by the Dagik Earth project (https://www.dagik.net/). The full animation is available at https://ads.nipr.ac.jp/node/dagik/?type=MIROC5/PERMAFROST_SEP

A web page showing an animation of future permafrost change (Fig. 12, https://ads.nipr.ac.jp/node/dagik/?type=MIROC5/PERMAFROST_SEP) has been created based on the future projection data that the authors provided to a research team at the National Institute of Polar Research specializing in such visualization (Arctic Data archive System). The animation was generated based on a tool developed by the Dagik Earth project (https://www.dagik.net/). On the created page, the center coordinates of the display (i.e., the angle of the projected map) can be freely changed by the viewer. With these animations, future projections indicating permafrost area reductions of as much as one-half are expressed in an easy-to-understand manner, not just for the general public, but for climate researchers as well.

## Data Availability

Data sharing is not applicable to this article as no datasets were generated or analyzed during the current study. Please contact the corresponding author for data requests.

## Abbreviations

ALT: Active layer thickness
CLAM: Circumpolar Active Layer Monitoring
CMIP: Coupled Model Inter-comparison Project
GCM: Global climate model
GHGs: Greenhouse gases
IPA: International Permafrost Association
ISIMIP: Inter-Sectoral Impact Model Inter-comparison Project
MATSIRO: Minimal Advanced Treatments of Surface Interaction and Runoff
MIROC: Model for Interdisciplinary Research on Climate
RCP: Representative Concentration Pathways
